# A Robust SMC-PHD Filter for Multi-Target Tracking with Unknown Heavy-Tailed Measurement Noise

**DOI:** 10.3390/s21113611

**Published:** 2021-05-22

**Authors:** Yang Gong, Chen Cui

**Affiliations:** Institute of Electronic Countermeasure, National University of Defense Technology, Hefei 230037, China; ky13285650152@163.com

**Keywords:** multi-target tracking, SMC-PHD filter, student-t distribution, variational Bayesian

## Abstract

In multi-target tracking, the sequential Monte Carlo probability hypothesis density (SMC-PHD) filter is a practical algorithm. Influenced by outliers under unknown heavy-tailed measurement noise, the SMC-PHD filter suffers severe performance degradation. In this paper, a robust SMC-PHD (RSMC-PHD) filter is proposed. In the proposed filter, Student-t distribution is introduced to describe the unknown heavy-tailed measurement noise where the degrees of freedom (DOF) and the scale matrix of the Student-t distribution are respectively modeled as a Gamma distribution and an inverse Wishart distribution. Furthermore, the variational Bayesian (VB) technique is employed to infer the unknown DOF and scale matrix parameters while the recursion estimation framework of the RSMC-PHD filter is derived. In addition, considering that the introduced Student- t distribution might lead to an overestimation of the target number, a strategy is applied to modify the updated weight of each particle. Simulation results demonstrate that the proposed filter is effective with unknown heavy-tailed measurement noise.

## 1. Introduction

Multi-target tracking (MTT) involves the estimate of target number and states from noisy measurements [1]. Influenced by clutter and detection uncertainty, the traditional MTT algorithms based on data association, such as joint probabilistic data association (JPDA) [2] and multiple hypotheses tracking (MHT) [3], are burdened with high computation complexity with the increase of target number and clutter number. Under the framework of finite set statistics (FISST), multi-target states and measurements are modeled as random finite sets (RFS) to avoid data association. Unfortunately, the MTT approaches based on RFS are still computationally intractable because of the multiple integrals. To obtain a computational tractable solution, the probability hypothesis density (PHD) filter [4] which propagates the first order moment approximation of multi-target posterior density is proposed and realizes the estimate of multi-target number and states. The two main implementations of the PHD filter are the Gaussian mixture PHD (GM-PHD) filter [5] and the sequential Monte Carle PHD (SMC-PHD) [6]. In recent years, the PHD filter has generated substantial interest in visual tracking [7], radar tracking [8], etc.

However, the above algorithms only achieve good performance with the known Gaussian measurement noise variance. When the measurement noise variance is unknown and inconsistent with the true variance, the performance of these algorithms decreases. In [9], Sarkka considers the unknown measurement noise variance problem under the linear Gaussian system and models noise variance as an inverse Gamma (IG) distribution. Then, the joint estimate of target state and noise variance is obtained by variational Bayesian (VB) technique. Since the inverse Gamma distribution is only suitable for the situation where the noise variance is diagonal, the inverse Wishart (IW) distribution is further used to model the full noise variance [10,11]. Following this, the VB technique was introduced into the PHD filter [12,13,14] for MTT.

In some applications, the measurement noise might be non-Gaussian. For example, due to the electromagnetic interference and the sensor’s unreliability, the measurement model is usually accompanied with outliers and the measurement noise can be treated as non-Gaussian noise with a heavy-tailed probability density function [15]. The outliers will result in the performance degradation of conventional tracking algorithms. In [15], the combination of a Laplacian distribution and a Gaussian distribution is used to describe the heavy-tailed measurement noise. In [16,17], the heavy-tailed measurement noise is characterized as a mixture of two Gaussian distributions. Due to the advantage of the heavy-tailed characteristic, Student-t distribution was widely used to deal with outliers in [18,19,20]. However, the relevant parameters of Student-t distribution need to be known in these applications. Similar to [9,10,11,12,13,14], the VB technique was employed in the situation where the Student-t distribution parameters are unknown in [21,22,23,24]. The joint estimate of target and unknown parameters is obtained in a linear system. However, the above approaches work worse in a nonlinear system. The nonlinear transformation algorithms, such as extended Kalman filter (EKF) and unscented Kalman filter (UKF), can be used to cope with the nonlinear system to some extent. Unfortunately, the EKF depends on the nature of nonlinearities and the UKF relies on the Gaussian characteristic of the system. They often give an unreliable estimate if the nonlinearities are severe or the system is non-Gaussian, which leads to target missing.

With heavy-tailed measurement noise, the robust PHD filter in a nonlinear system has been much less studied than that in a linear system. Compared with nonlinear transformations, the particle filter is more suitable for practical applications because it can deal with nonlinear and non-Gaussian dynamics. Motivated by [23], we extend the Student-t distribution into SMC-PHD filter and propose a robust SMC-PHD (RSMC-PHD) filter with unknown heavy-tailed measurement noise. In the proposed filter, the Student-t distribution where the degrees of freedom (DOF) and the scale matrix are both unknown is used to model the measurement noise. The Gamma distribution and IW distribution are respectively used to model the DOF and the scale matrix. Then, the augmented state of the target involves the target state, DOF, and the scale matrix. Further, the target state is estimated by particle approximations and the DOF and the scale matrix are updated by the VB technique within the SMC-PHD filter framework. In addition, considering the problem that the introduced Student-t distribution might lead to an overestimation of the target number, we apply a strategy to modify the updated weight of each particle. Simulation results show that the proposed RSMC-PHD filter can achieve higher estimate accuracy than the compared filter under unknown heavy-tailed measurement noise.

This paper is organized as follows. Section 2 gives a brief overview of the tracking model, measurement noise model, PHD filter, and SMC-PHD filter. Section 3 presents the detailed implementation of the proposed filter. Section 4 evaluates the performance of the proposed filter under two scenarios. Finally, Section 5 displays the conclusions.

## 2. Background

### 2.1. Tracking Model and Measurement Noise Model

Consider a state space system given by

(1) $$x_{k}=f(x_{k-1})+w_{k}$$

(2) $$z_{k}=h(x_{k})+\varepsilon_{k}$$

where $x_{k}$ is referred to as the target state and $z_{k}$ is referred to as the measurement at time k. $w_{k}$ is the process noise assumed to be Gaussian white noise with covariance matrix $Q_{k}$. f(⋅) and h(⋅) are the dynamic motion model function and measurement model function, respectively. $\varepsilon_{k}$ is the measurement noise.

The target dynamic motion model function is given by

(3) $$f\left(x_{k}\left|x_{k-1}\right.\right)=N\left(x_{k};f\left(x_{k-1}\right),Q_{k}\right)$$

where $N\left(x;\hat{x},P\right)$ denotes a Gaussian probability density function with mean $\hat{x}$ and covariance matrix P. Assume the measurement noise has a heavy tail and can be described by a Student-t distribution as [18]

(4) $$\begin{array}{cc} p(\varepsilon_{k}) & =\mathrm{St}(\varepsilon_{k};0_{d},R_{k},\nu_{k})\\ & =\frac{\Gamma \left(\frac{\nu_{k}+d}{2}\right)}{\Gamma \left(\frac{\nu_{k}}{2}\right)}\frac{1}{{\left(\nu_{k}\pi \right)}^{\frac{d}{2}}}\frac{1}{{\left|R_{k}\right|}^{\frac{1}{2}}}{\left(1+\frac{1}{\nu_{k}}\varepsilon_{k}^{T}R_{k}{}^{-1}\varepsilon_{k}\right)}^{-\frac{\nu_{k}+d}{2}} \end{array}$$

where St(⋅) denotes the Student-t distribution, $0_{d}$ denotes d dimension zeros vector, d is the dimension of $z_{k}$, $\nu_{k}$ denotes DOF, $R_{k}$ is a symmetric, positive defined d×d covariance matrix and Γ(⋅) denotes the Gamma function. Different from the Gaussian distribution, the Student-t distribution has a thicker tail away from the mean value. When $\nu_{k}\to \infty$, the Student-t distribution is equivalent to a Gaussian distribution. The probability density function in Equation (4) can be also formulated as a mixture of Gamma distribution and Gaussian distribution denoted as [25]

(5) $$\mathrm{St}(\varepsilon_{k};0,R_{k},\nu_{k})=\int_{0}^{\infty }N\left(\varepsilon_{k};0,R_{k}/u_{k}\right)G\left(u_{k};\frac{\nu_{k}}{2},\frac{\nu_{k}}{2}\right)du_{k}$$

where $G\left(\cdot \right)$ denotes the gamma distribution, $u_{k}$ is an auxiliary variable. The likelihood of the measurement can be written as

(6) $$g_{k}(z_{k}\left|x_{k},R_{k},\nu_{k}\right.)=\mathrm{St}(z_{k};h(x_{k}),R_{k},\nu_{k})$$

According to Equation (5), we can obtain

(7) $$g_{k}(z_{k}\left|x_{k},R_{k},\mu_{k}\right.)=N\left(z_{k};h(x_{k}),R_{k}/u_{k}\right)$$

where $u_{k}$ is distributed as

(8) $$p(u_{k}\left|\nu_{k}\right.)=G\left(u_{k};\nu_{k}/2,\nu_{k}/2\right)$$

In this paper, the DOF $\nu_{k}$ and scale matrix $R_{k}$ are both unknown. Considering the conjugate prior to Gaussian distribution with unknown variance is the IW distribution, we model $R_{k}$ as the IW distribution. Similarly, the Gamma distribution is assumed to be the conjugate prior to the DOF $\nu_{k}$. Then the joint probability density of $R_{k}$, $u_{k}$, and $\nu_{k}$ can be expressed as

(9) $$\begin{array}{cc} p\left(R_{k},u_{k},\nu_{k}\right) & =\mathrm{IW}\left(R_{k};\upsilon_{k},V_{k}\right)G\left(u_{k};\nu_{k}/2,\nu_{k}/2\right)\\ & \times G\left(\nu_{k};\alpha_{k},\beta_{k}\right) \end{array}$$

where IW(⋅) denotes the IW distribution, $\upsilon_{k}$, $V_{k}$ are the sufficient statistics of $R_{k}$, $\alpha_{k}$ and $\beta_{k}$ are the sufficient statistics of $\nu_{k}$.

### 2.2. PHD Filter

Assume that $X_{k}=\{x_{k}^{1},\cdots ,x_{k}^{N_{k}}\}$ is the multi-target states set and $Z_{k}=\{z_{k}^{1},\cdots ,z_{k}^{M_{k}}\}$ is the measurements set at time k. $x_{k}^{n}$ is referred to as the nth target state and $z_{k}^{m}$ is referred to as the mth measurement. $N_{k}$ and $M_{k}$ are the number of targets and measurements, respectively. Then the Bayesian filter recursion can be expressed as

(10) $$p_{k\left|k-1\right.}(X_{k}\left|Z_{1:k-1}\right.)=\int f(X_{k}\left|X_{k-1}\right.)p_{k-1}(X_{k-1}\left|Z_{1:k-1}\right.)\mu_{s}dX_{k-1}$$

(11) $$p_{k}(X_{k}\left|Z_{1:k}\right.)=\frac{g_{k}(Z_{k}\left|X_{k}\right.)p_{k\left|k-1\right.}(X_{k}\left|Z_{1:k-1}\right.)}{\int g_{k}(Z_{k}\left|X_{k}\right.)p_{k\left|k-1\right.}(X_{k}\left|Z_{1:k-1}\right.)\mu_{s}dX_{k}}$$

where $g_{k}(\cdot )$ is the likelihood function. $p_{k\left|k-1\right.}(\cdot )$ and $p_{k}(\cdot )$ denote multi-target predicted density and multi-target posterior density, respectively. $\mu_{s}$ is the reference measure.

The recursion Equations (10) and (11) involve multiple integrals, which are computationally intractable. To alleviate the computational intractability, the PHD filter provides a sub-optimal solution by propagating the first-order statistical moment of the multiple posterior densities. Without the spawning targets, the predicted and updated equations of the PHD filter are given by [5]

(12) $$D_{k\left|k-1\right.}(x_{k})=\int p_{S,k}f(x_{k}\left|x_{k-1}\right.)D_{k-1}(x_{k-1})dx_{k-1}+\gamma_{k}(x_{k})$$

(13) $$D_{k}(x_{k})=(1-p_{D,k})D_{k\left|k-1\right.}(x_{k})+\sum_{z\in Z_{k}}\frac{p_{D,k}g_{k}(z\left|x_{k}\right.)D_{k\left|k-1\right.}(x_{k})}{\kappa_{k}(z)+\int p_{D,k}g_{k}(z\left|x_{k}\right.)D_{k\left|k-1\right.}(x_{k})dx_{k}}$$

where $D_{k\left|k-1\right.}(x_{k})$ and $D_{k\left|k\right.}(x_{k})$ are the predicted intensity and posterior intensity, respectively. $\gamma_{k}(x_{k})$ is the birth targets intensity, $p_{S,k}$ is the target survival probability, $p_{D,k}$ is the detection probability. $\kappa_{k}(z)=\lambda c(z)$ is the clutter intensity with average clutter number λ and spatial distribution c(z).

### 2.3. SMC-PHD Filter

Assume that the multi-target posterior intensity can be denoted by weighted particles set at time k−1, i.e., $D_{k-1}(x_{k-1}\left|Z_{1:k-1}\right.)=\sum_{i=1}^{L_{k-1}}\omega_{k-1}^{\left(i\right)}\delta (x_{k-1}-x_{k-1}^{\left(i\right)})$. $L_{k-1}$ denotes the particle number at time k−1, $x_{k-1}^{\left(i\right)}$ is the *i*th particle state and $\omega_{k-1}^{\left(i\right)}$ is the corresponding weight. Then, the SMC-PHD filter can be summarized by the following steps.

Step 1 Prediction

(14) $$x_{k}^{\left(i\right)}∼\left\{\begin{array}{l} q_{k}(\cdot \left|x_{k-1}^{\left(i\right)},Z_{k}\right.),i=1,\ldots ,L_{k-1}\\ p_{k}(\cdot \left|Z_{k}\right.),i=L_{k-1}+1,\ldots ,L_{k-1}+J_{k} \end{array}\right.$$

(15) $$\omega_{k\left|k-1\right.}^{\left(i\right)}∼\left\{\begin{array}{l} \omega_{k-1}^{\left(i\right)}\frac{p_{S,k}f_{k\left|k-1\right.}(x_{k}^{\left(i\right)}\left|x_{k-1}^{\left(i\right)}\right.)}{q_{k}(x_{k}^{\left(i\right)}\left|x_{k-1}^{\left(i\right)},Z_{k}\right.)},i=1,\ldots ,L_{k-1}\\ \frac{1}{J_{k}}\frac{\gamma_{k}(x_{k}^{\left(i\right)})}{p_{k}(x_{k}^{\left(i\right)}\left|Z_{k}\right.)},i=L_{k-1}+1,\ldots ,L_{k-1}+J_{k} \end{array}\right.$$

(16) $$D_{k\left|k-1\right.}(x_{k}\left|Z_{1:k-1}\right.)=\sum_{i=1}^{L_{k-1}+J_{k}}\omega_{k\left|k-1\right.}^{\left(i\right)}\delta (x_{k}-x_{k}^{\left(i\right)})$$

where $q_{k}\left(\cdot \left|x_{k-1}^{\left(i\right)},Z_{k}\right.\right)$ is the proposal density of survival targets and $p_{k}\left(\cdot \left|Z_{k}\right.\right)$ is the proposal density of birth targets. $J_{k}$ is the particles number of birth targets.

Step 2 Update

(17) $$\omega_{k}^{\left(i\right)}=(1-p_{D,k})\omega_{k\left|k-1\right.}^{\left(i\right)}+\sum_{z\in Z_{k}}\frac{p_{D,k}g_{k}(z\left|x_{k}^{\left(i\right)}\right.)}{\kappa_{k}(z)+C_{k}(z)}\omega_{k\left|k-1\right.}^{\left(i\right)}$$

(18) $$C_{k}(z)=\sum_{j=1}^{L_{k-1}+J_{k}}p_{D,k}g_{k}(z\left|x_{k}^{\left(i\right)}\right.)\omega_{k\left|k-1\right.}^{\left(i\right)}$$

(19) $$D_{k}(x_{k}\left|Z_{1:k}\right.)=\sum_{i=1}^{L_{k-1}+J_{k}}\omega_{k}^{\left(i\right)}\delta (x_{k}-x_{k}^{\left(i\right)})$$

Step 3 Resampling

Target number is estimated by ${\hat{N}}_{k}=\mathrm{ceil}\left(\sum_{i=1}^{L_{k-1}+J_{k}}\omega_{k}^{\left(i\right)}\right)$, and resample ${\left\{x_{k}^{\left(i\right)},\omega_{k}^{\left(i\right)}\right\}}_{i=1}^{L_{k-1}+J_{k}}$. ceil(x) denotes the smallest integer more than x, $L_{k}=N_{particle}\cdot {\hat{N}}_{k}$, $N_{particle}$ is the number of particles per expected target.

Step 4 State extraction

Extract the target states $X_{k}=\{{\hat{x}}_{k}^{1},\cdots ,{\hat{x}}_{k}^{{\hat{N}}_{k}}\}$ from the resampling particles set, where ${\hat{x}}_{k}^{1},\cdots ,{\hat{x}}_{k}^{{\hat{N}}_{k}}$ denote the estimated target states.

## 3. The Robust SMC-PHD Filter

### 3.1. VB Approximation

The aforementioned SMC-PHD filter performs well under Gaussian measurement noise. However, the outliers in measurements will have a negative effect on the SMC-PHD filter. To solve the problem, the Student-t distribution is introduced to model the heavy-tailed measurement noise. Since the parameters of the Student-t distribution are unknown, the joint posterior probability density of target state $x_{k}$, scale matrix $R_{k}$, auxiliary variable $u_{k}$, and DOF $\nu_{k}$ need to be estimated. Therefore, the augmented target state can be expressed as

(20) $${\ddot{x}}_{k}=\left(x_{k},R_{k},u_{k},\nu_{k}\right)$$

Since there is no explicit analytical solution existing for the joint posterior density $p({\ddot{x}}_{k}\left|Z_{1:k}\right.)$, we utilize the VB inference [9] to obtain the approximate solution. As for an approximate method, VB inference is widely used in searching for a factored approximate posterior density as follows

(21) $$\begin{array}{cc} p({\ddot{x}}_{k}\left|Z_{1:k}\right.) & =p(x_{k},R_{k},u_{k},\nu_{k}\left|Z_{1:k}\right.)\\ & \approx q({\ddot{x}}_{k}\left|Z_{1:k}\right.)≜q_{x}(x_{k})q_{R}(R_{k})q_{u}(u_{k})q_{v}(\nu_{k}) \end{array}$$

where $q({\ddot{x}}_{k}\left|Z_{1:k}\right.)$ is the approximate joint posterior density, $q_{x}(\cdot )$, $q_{R}(\cdot )$, $q_{u}(\cdot )$,and $q_{\nu }(\cdot )$ are the approximate posterior densities. Equation (21) has the implicit assumption that the target state and the Student-t distribution parameters are independent. By minimizing the Kullback–Leibler (KL) divergence between $p({\ddot{x}}_{k}\left|Z_{1:k}\right.)$ and $q({\ddot{x}}_{k}\left|Z_{1:k}\right.)$, the estimate ${\hat{q}}_{x}(x_{k})$, ${\hat{q}}_{R}(R_{k})$, ${\hat{q}}_{u}(u_{k})$, and ${\hat{q}}_{\nu }(\nu_{k})$ can be easily obtained by

(22) $$\left\{{\hat{q}}_{x},{\hat{q}}_{R},{\hat{q}}_{u},{\hat{q}}_{\nu }\right\}=\underset{q_{x},q_{R},q_{u},q_{\nu }}{\arg\min}KL\left[q(\left\{{\ddot{x}}_{k}\right\}\left|Z_{1:k}\right.)\left\|p(\left\{{\ddot{x}}_{k}\right\}\left|Z_{1:k}\right.)\right.\right]$$

where $KL(q(x)\left|p(x)\right.)≜\int q(x)\log\left(\frac{q(x)}{p(x)}\right)dx$. The optimal solution of Equation (22) can be obtained by [9,21,26]

(23) $$\ln\hat{q}(\phi )=E_{{\ddot{x}}_{k}^{\left(-\phi \right)}}\left[\ln p\left({\ddot{x}}_{k},Z_{k}\left|Z_{1:k-1}\right.\right)\right]+c_{\phi }$$

where ϕ denotes an arbitrary element in ${\ddot{x}}_{k}$, E[⋅] denotes the expectation, ${\ddot{x}}_{k}^{\left(-\phi \right)}$ denotes the elements set in ${\ddot{x}}_{k}$ except for ϕ, and $c_{\phi }$ is an irrelevant constant for element ϕ.

### 3.2. The Update of Unknown Student-t Distribution Parameters

Based on the assumption that the target state is independent of the Student-t distribution parameters, the joint posterior density of the augmented target state can be factorized as

(24) $$p({\ddot{x}}_{k}\left|Z_{1:k}\right.)=p(R_{k},u_{k},\nu_{k}\left|Z_{1:k}\right.)p(x_{k}\left|Z_{1:k}\right.)$$

where the unknown Student-t distribution parameters are integrated out. Based on (24), the target state can be estimated by particle samples and the Student-t distribution parameters can be updated by the VB approximation method.

In this subsection, we derive the update of Student-t distribution parameters for each particle. At time k, let ${\ddot{x}}_{k}=\{{\ddot{x}}_{k}^{\left(i\right)}{\}}_{i=1}^{N}=\{x_{k}^{\left(i\right)},R_{k}^{\left(i\right)},u_{k}^{\left(i\right)},\nu_{k}^{\left(i\right)}{\}}_{i=1}^{N}$ is the augmented target state where $R_{k}^{\left(i\right)}$, $u_{k}^{\left(i\right)}$, $\nu_{k}^{\left(i\right)}$ are the Student-t distribution parameters with respect to the ith particle, and N is the number of particles. Assume the predicted density of ${\ddot{x}}_{k}^{\left(i\right)}$ at time k can be expressed as

(25) $$\begin{array}{cc} p({\ddot{x}}_{k}^{\left(i\right)}\left|Z_{1:k-1}\right.) & =\omega_{k\left|k-1\right.}^{\left(i\right)}\delta (x_{k}-x_{k}^{\left(i\right)})\mathrm{IW}(R_{k}^{\left(i\right)};\upsilon_{k\left|k-1\right.}^{\left(i\right)},V_{k\left|k-1\right.}^{\left(i\right)})\\ & \times G\left(u_{k}^{\left(i\right)};\frac{\nu_{k}^{\left(i\right)}}{2},\frac{\nu_{k}^{\left(i\right)}}{2}\right)G(\nu_{k}^{\left(i\right)};\alpha_{k\left|k-1\right.}^{\left(i\right)},\beta_{k\left|k-1\right.}^{\left(i\right)}) \end{array}$$

where $\upsilon_{k\left|k-1\right.}^{\left(i\right)}$, $V_{k\left|k-1\right.}^{\left(i\right)}$, $\alpha_{k\left|k-1\right.}^{\left(i\right)}$ and $\beta_{k\left|k-1\right.}^{\left(i\right)}$ are the predicted sufficient statistics with respect to $R_{k}^{\left(i\right)}$ and $\nu_{k}^{\left(i\right)}$. Then the joint density of $p({\ddot{x}}_{k}^{\left(i\right)},z_{k}\left|Z_{1:k-1}\right.)$ can be expressed as

(26) $$\begin{array}{cc} p({\ddot{x}}_{k}^{\left(i\right)},z_{k}\left|Z_{1:k-1}\right.)= & \omega_{k\left|k-1\right.}^{\left(i\right)}\delta (x_{k}-x_{k}^{\left(i\right)})N\left(z_{k};h(x_{k}^{\left(i\right)}),\frac{R_{k}^{\left(i\right)}}{u_{k}^{\left(i\right)}}\right)\\ & \times \mathrm{IW}(R_{k}^{\left(i\right)};\upsilon_{k\left|k-1\right.}^{\left(i\right)},V_{k\left|k-1\right.}^{\left(i\right)})G\left(u_{k}^{\left(i\right)};\frac{\nu_{k}^{\left(i\right)}}{2},\frac{\nu_{k}^{\left(i\right)}}{2}\right)\\ & \times G(\nu_{k}^{\left(i\right)};\alpha_{k\left|k-1\right.}^{\left(i\right)},\beta_{k\left|k-1\right.}^{\left(i\right)}) \end{array}$$

where $z_{k}$ is the measurement with respect to the target represented by particle $x_{k}^{\left(i\right)}$. Based on Equation (26), $\ln p({\ddot{x}}_{k}^{\left(i\right)},z_{k}\left|Z_{1:k-1}\right.)$ can be expressed as

(27) $$\begin{array}{cc} \ln p({\ddot{x}}_{k}^{\left(i\right)},z_{k}\left|Z_{1:k-1}\right.) & =-\frac{1}{2}\ln\left|R_{k}^{\left(i\right)}\right|+\frac{d}{2}\ln u_{k}^{\left(i\right)}-\frac{u_{k}^{\left(i\right)}}{2}tr\left({\left(R_{k}^{\left(i\right)}\right)}^{-1}\Xi_{k}^{\left(i\right)}\right)-\frac{\upsilon_{k\left|k-1\right.}^{\left(i\right)}}{2}\ln\left|R_{k}^{\left(i\right)}\right|\\ & -\frac{1}{2}tr\left({\left(R_{k}^{\left(i\right)}\right)}^{-1}V_{k\left|k-1\right.}^{\left(i\right)}\right)+\frac{\nu_{k}^{\left(i\right)}}{2}\ln\frac{\nu_{k}^{\left(i\right)}}{2}-\ln\Gamma (\frac{\nu_{k}^{\left(i\right)}}{2})+(\frac{\nu_{k}^{\left(i\right)}}{2}-1)\ln u_{k}^{\left(i\right)}\\ & -\frac{\nu_{k}^{\left(i\right)}}{2}u_{k}^{\left(i\right)}+(\alpha_{k\left|k-1\right.}^{\left(i\right)}-1)\ln\nu_{k}^{\left(i\right)}-\beta_{k\left|k-1\right.}^{\left(i\right)}\nu_{k}^{\left(i\right)}+c_{{\ddot{x}}_{k}^{\left(i\right)}} \end{array}$$

where $\Xi_{k}^{\left(i\right)}=(z_{k}-h(x_{k}^{\left(i\right)})){\left(z_{k}-h(x_{k}^{\left(i\right)})\right)}^{T}$ denotes the innovation covariance matrix. tr(⋅) denotes the trace operation. $c_{{\ddot{x}}_{k}^{\left(i\right)}}$ denotes an irrelevant constant for variables $x_{k}^{\left(i\right)}$, $R_{k}^{\left(i\right)}$, $u_{k}^{\left(i\right)}$, and $\nu_{k}^{\left(i\right)}$.

Letting $\phi =R_{k}^{\left(i\right)}$ and using Equation (27) in Equation (23), we can obtain

(28) $$\ln\hat{q}(R_{k}^{\left(i\right)})=-\frac{\upsilon_{k\left|k-1\right.}^{\left(i\right)}+1}{2}\ln\left|R_{k}^{\left(i\right)}\right|-\frac{1}{2}\left\{tr\left({\left(R_{k}^{\left(i\right)}\right)}^{-1}\left[V_{k\left|k-1\right.}^{\left(i\right)}+u_{k}^{\left(i\right)}\Xi_{k}^{\left(i\right)}\right]\right)\right\}+c_{R_{k}^{\left(i\right)}}$$

The posterior distribution $\hat{q}(R_{k}^{\left(i\right)})$ of $R_{k}^{\left(i\right)}$ is an IW distribution, i.e.,

(29) $$\hat{q}(R_{k}^{\left(i\right)})=\mathrm{IW}(R_{k}^{\left(i\right)};\upsilon_{k}^{\left(i\right)},V_{k}^{\left(i\right)})$$

with parameters

(30) $$\upsilon_{k}^{\left(i\right)}=\upsilon_{k\left|k-1\right.}^{\left(i\right)}+1$$

(31) $$V_{k}^{\left(i\right)}=V_{k\left|k-1\right.}^{\left(i\right)}+E[u_{k}^{\left(i\right)}]\Xi_{k}^{\left(i\right)}$$

Letting $\phi =\nu_{k}^{\left(i\right)}$ and using Equation (27) in Equation (23), we can obtain

(32) $$\begin{array}{cc} \ln\hat{q}(\nu_{k}^{\left(i\right)}) & =\frac{\nu_{k}^{\left(i\right)}}{2}\ln\frac{\nu_{k}^{\left(i\right)}}{2}-\ln\Gamma (\frac{\nu_{k}^{\left(i\right)}}{2})+\frac{\nu_{k}^{\left(i\right)}}{2}\ln u_{k}^{\left(i\right)}\\ & -\frac{\nu_{k}^{\left(i\right)}}{2}u_{k}^{\left(i\right)}+(\alpha_{k\left|k-1\right.}^{\left(i\right)}-1)\ln\nu_{k}^{\left(i\right)}-\beta_{k\left|k-1\right.}^{\left(i\right)}\nu_{k}^{\left(i\right)}+c_{\nu_{k}^{\left(i\right)}} \end{array}$$

Using Stirling’s approximation in [27]

(33) $$\ln\Gamma (\frac{\nu_{k}^{\left(i\right)}}{2})\approx \frac{\nu_{k}^{\left(i\right)}-1}{2}\ln\frac{\nu_{k}^{\left(i\right)}}{2}-\frac{\nu_{k}^{\left(i\right)}}{2}$$

And $\ln\hat{q}(\nu_{k}^{\left(i\right)})$ can be expressed as

(34) $$\begin{array}{cc} \ln\hat{q}(\nu_{k}^{\left(i\right)}) & =(\alpha_{k\left|k-1\right.}^{\left(i\right)}+\frac{1}{2}-1)\ln\nu_{k}^{\left(i\right)}\\ & -(\beta_{k\left|k-1\right.}^{\left(i\right)}-\frac{1}{2}(1+E[\ln u_{k}^{\left(i\right)}]-E[u_{k}^{\left(i\right)}]))\nu_{k}^{\left(i\right)} \end{array}$$

The posterior distribution $\hat{q}(\nu_{k}^{\left(i\right)})$ of $\nu_{k}^{\left(i\right)}$ is a Gamma distribution, i.e.,

(35) $$\hat{q}(\nu_{k}^{\left(i\right)})=G(\nu_{k}^{\left(i\right)};\alpha_{k}^{\left(i\right)},\beta_{k}^{\left(i\right)})$$

with parameters

(36) $$\alpha_{k}^{\left(i\right)}=\alpha_{k\left|k-1\right.}^{\left(i\right)}+\frac{1}{2}$$

(37) $$\beta_{k}^{\left(i\right)}=\beta_{k\left|k-1\right.}^{\left(i\right)}-\frac{1}{2}(1+E[\ln u_{k}^{\left(i\right)}]-E[u_{k}^{\left(i\right)}])$$

Letting $\phi =u_{k}^{\left(i\right)}$ and using Equation (27) in Equation (23), we can obtain

(38) $$\ln\hat{q}(u_{k}^{\left(i\right)})=(\frac{\nu_{k}^{\left(i\right)}+d}{2}-1)\ln u_{k}^{\left(i\right)}-\frac{1}{2}[\nu_{k}^{\left(i\right)}+tr\left({\left(R_{k}^{\left(i\right)}\right)}^{-1}\Xi_{k}^{\left(i\right)}\right)]u_{k}^{\left(i\right)}+c_{u_{k}^{\left(i\right)}}$$

The posterior distribution $\hat{q}(u_{k}^{\left(i\right)})$ of $u_{k}^{\left(i\right)}$ is a Gamma distribution, i.e.,

(39) $$\hat{q}(u_{k}^{\left(i\right)})=G(u_{k}^{\left(i\right)};\frac{\nu_{k}^{\left(i\right)}+d}{2},\frac{1}{2}[\nu_{k}^{\left(i\right)}+tr\left({\left(R_{k}^{\left(i\right)}\right)}^{-1}\Xi_{k}^{\left(i\right)}\right)])$$

Then we can obtain

(40) $$E[u_{k}^{\left(i\right)}]=a_{k}^{\left(i\right)}/b_{k}^{\left(i\right)}$$

(41) $$E[\ln u_{k}^{\left(i\right)}]=\varphi (a_{k}^{\left(i\right)})-\ln b_{k}^{\left(i\right)}$$

where $a_{k}^{\left(i\right)}=\frac{E[\nu_{k}^{\left(i\right)}]+d}{2}$, $b_{k}^{\left(i\right)}=\frac{1}{2}\left\{E[\nu_{k}^{\left(i\right)}]+tr\left({\left(R_{k}^{\left(i\right)}\right)}^{-1}\Xi_{k}^{\left(i\right)}\right)\right\}$. Since the parameters in Equations (30), (31), (36), (37), (40) and (41) are coupled, it can be solved iteratively by the fixed point method in [21].

### 3.3. The Implementation of RSMC-PHD Filter

In this subsection, we introduce the Student-t distribution into the SMC-PHD filter and present an implementation of the robust SMC-PHD filter with unknown heavy-tailed measurement noise. Assume that the multi-target posterior intensity is represented by a weighted particles set ${\left\{\omega_{k-1}^{\left(i\right)},x_{k-1}^{\left(i\right)},R_{k-1}^{\left(i\right)},u_{k-1}^{\left(i\right)},\nu_{k-1}^{\left(i\right)}\right\}}_{i=1}^{L_{k-1}}$ at time k−1, i.e.,

(42) $$\begin{array}{cc} D_{k-1}({\ddot{x}}_{k-1}\left|Z_{1:k-1}\right.) & =\sum_{i=1}^{L_{k-1}}\omega_{k-1}^{\left(i\right)}\delta (x_{k-1}-x_{k-1}^{\left(i\right)})\mathrm{IW}(R_{k-1}^{\left(i\right)};\upsilon_{k-1}^{\left(i\right)},V_{k-1}^{\left(i\right)})\\ & \times G\left(u_{k}^{\left(i\right)};\frac{\nu_{k-1}^{\left(i\right)}}{2},\frac{\nu_{k-1}^{\left(i\right)}}{2}\right)G(\nu_{k-1}^{\left(i\right)};\alpha_{k-1}^{\left(i\right)},\beta_{k-1}^{\left(i\right)}) \end{array}$$

Assume $R_{k}$, $u_{k}$, and $\nu_{k}$ are independent and the predicted intensity at time k can be expressed as

(43) $$\begin{array}{cc} D_{k\left|k-1\right.}({\ddot{x}}_{k}\left|Z_{1:k-1}\right.) & =\sum_{i=1}^{L_{k-1}+J_{k}}\omega_{k\left|k-1\right.}^{\left(i\right)}\delta (x_{k}-x_{k}^{\left(i\right)})G\left(u_{k}^{\left(i\right)};\frac{\nu_{k}^{\left(i\right)}}{2},\frac{\nu_{k}^{\left(i\right)}}{2}\right)\\ & \times \mathrm{IW}(R_{k}^{\left(i\right)};\upsilon_{k\left|k-1\right.}^{\left(i\right)},V_{k\left|k-1\right.}^{\left(i\right)})G(\nu_{k}^{\left(i\right)};\alpha_{k\left|k-1\right.}^{\left(i\right)},\beta_{k\left|k-1\right.}^{\left(i\right)}) \end{array}$$

where $\omega_{k\left|k-1\right.}^{\left(i\right)}$ and $x_{k}^{\left(i\right)}$ are given in Equations (14) and (15). There are $L_{k-1}$ particles for survival targets and $J_{k}$ particles for birth targets. Generally, the dynamic of measurement noise is unknown and we use the similar heuristic dynamic in [9] for the predicted sufficient statistics. For the particles of survival targets $i=1,2,\ldots ,L_{k-1}$, the predicted sufficient statistics are given by

(44) $$\upsilon_{k\left|k-1\right.}^{\left(i\right)}=\rho (\upsilon_{k-1}^{\left(i\right)}-d-1)+d+1,V_{k\left|k-1\right.}^{\left(i\right)}=\rho V_{k-1}^{\left(i\right)}$$

(45) $$\alpha_{k\left|k-1\right.}^{\left(i\right)}=\rho \alpha_{k-1}^{\left(i\right)},\beta_{k\left|k-1\right.}^{\left(i\right)}=\rho \beta_{k-1}^{\left(i\right)}$$

where ρ∈(0,1] is the decreasing factor. For the particles of birth targets $i=L_{k-1}+1,L_{k-1}+2,\ldots ,L_{k-1}+J_{k}$, the predicted sufficient statistics are given by

(46) $$\upsilon_{k\left|k-1\right.}^{\left(i\right)}=\upsilon_{\gamma ,k}^{\left(i\right)},V_{k\left|k-1\right.}^{\left(i\right)}=V_{\gamma ,k}^{\left(i\right)}$$

(47) $$\alpha_{k\left|k-1\right.}^{\left(i\right)}=\rho \alpha_{k-1}^{\left(i\right)},\beta_{k\left|k-1\right.}^{\left(i\right)}=\rho \beta_{k-1}^{\left(i\right)}$$

where $\upsilon_{\gamma ,k}^{\left(i\right)}$, $V_{\gamma ,k}^{\left(i\right)}$, $\alpha_{\gamma ,k}^{\left(i\right)}$ and $\beta_{\gamma ,k}^{\left(i\right)}$ are sufficient statistics with respect to the birth targets.

The posterior intensity can be expressed as

(48) $$\begin{array}{cc} D_{k}({\ddot{x}}_{k}\left|Z_{1:k}\right.) & =\sum_{i=1}^{L_{k-1}+J_{k}}\omega_{k}^{\left(i\right)}\delta (x_{k}-x_{k}^{\left(i\right)})G\left(u_{k}^{\left(i\right)};\frac{\nu_{k}^{\left(i\right)}}{2},\frac{\nu_{k}^{\left(i\right)}}{2}\right)\\ & \times \mathrm{IW}(R_{k}^{\left(i\right)};\upsilon_{k}^{\left(i\right)},V_{k}^{\left(i\right)})G(\nu_{k}^{\left(i\right)};\alpha_{k}^{\left(i\right)},\beta_{k}^{\left(i\right)}) \end{array}$$

where $\omega_{k}^{\left(i\right)}$ is given in (17). It is worth mentioning that the likelihood function is $g_{k}(z\left|{\ddot{x}}_{k}\right.)=\mathrm{St}(z;h(x_{k}^{\left(i\right)}),R_{k}^{\left(i\right)},\nu_{k}^{\left(i\right)})$. Then, the sufficient statistics $\upsilon_{k}^{\left(i\right)}$, $V_{k}^{\left(i\right)}$, $\alpha_{k}^{\left(i\right)}$, and $\beta_{k}^{\left(i\right)}$ are updated by

(49) $$R_{k}^{\left(i)(j\right)}=V_{k}^{\left(i)(j-1\right)}/\left(\upsilon_{k}^{\left(i\right)}-d-1\right)$$

(50) $${\tilde{R}}_{k}^{\left(i)(j\right)}=R_{k}^{\left(i)(j\right)}/u_{k}^{\left(i)(j-1\right)}$$

(51) $$\gamma_{k}^{\left(i)(j\right)}=tr(\Xi_{k}^{\left(i\right)}/{\tilde{R}}_{k}^{\left(i)(j\right)})$$

(52) $$a_{k}^{\left(i)(j\right)}=\left(\nu_{k}^{\left(i)(j-1\right)}+d\right)/2$$

(53) $$b_{k}^{\left(i)(j\right)}=\left(\nu_{k}^{\left(i)(j-1\right)}+\gamma_{k}^{\left(i)(j\right)}\right)/2$$

(54) $$u_{k}^{\left(i)(j\right)}=a_{k}^{\left(i)(j\right)}/b_{k}^{\left(i)(j\right)}$$

(55) $$\beta_{k}^{\left(i)(j\right)}=\beta_{k\left|k-1\right.}^{\left(i\right)}-\frac{1}{2}-\frac{\varphi (a_{k}^{\left(i)(j\right)})-\ln(b_{k}^{\left(i)(j\right)})}{2}+\frac{u_{k}^{\left(i)(j\right)}}{2}$$

(56) $$V_{k}^{\left(i)(j\right)}=V_{k\left|k-1\right.}^{\left(i\right)}+\Xi_{k}^{\left(i\right)}u_{k}^{\left(i)(j\right)}$$

(57) $$\nu_{k}^{\left(i)(j\right)}=\alpha_{k}^{\left(i\right)}/\beta_{k}^{\left(i)(j\right)}$$

where ${\left(\cdot \right)}^{\left(i)(j\right)}$ denotes the update of parameter ${\left(\cdot \right)}^{\left(i\right)}$ at the *j*th iteration. The initial values are given by $V_{k}^{\left(i)(0\right)}=V_{k\left|k-1\right.}^{\left(i\right)}$, $\upsilon_{k}^{\left(i\right)}=\upsilon_{k\left|k-1\right.}^{\left(i\right)}+1$, $\alpha_{k}^{\left(i\right)}=\alpha_{k\left|k-1\right.}^{\left(i\right)}+1/2$, $\beta_{k}^{\left(i)(0\right)}=\beta_{k\left|k-1\right.}^{\left(i\right)}$, $\nu_{k}^{\left(i)(0\right)}=$$\alpha_{k}^{\left(i\right)}/\beta_{k}^{\left(i)(0\right)}$.

It should be noted that the innovation covariance matrix $\Xi_{k}^{\left(i\right)}=(z_{k}-h(x_{k}^{\left(i\right)})){\left(z_{k}-h(x_{k}^{\left(i\right)})\right)}^{T}$ for particle $x_{k}^{\left(i\right)}$ is needed in each iteration. Since there is no explicit association between measurements and targets, the measurement origin is ambiguous. Considering the fact that the measurement with larger likelihood value is more likely generated by a potential target, we choose the measurement with the largest likelihood value to calculate the innovation covariance matrix as follows

(58) $$\Xi_{k}^{\left(i\right)}=(z_{k}^{l}-h(x_{k}^{\left(i\right)})){\left(z_{k}^{l}-h(x_{k}^{\left(i\right)})\right)}^{T}$$

where $l=\underset{p\in I}{\arg\max}g_{k}^{p}({\ddot{x}}_{k}^{\left(i\right)})$, $I=\left\{1,\ldots ,\left|Z_{k}\right|\right\}$. $g_{k}^{p}({\ddot{x}}_{k}^{\left(i\right)})$ is the normalized likelihood function value given by

(59) $$g_{k}^{p}({\ddot{x}}_{k}^{\left(i\right)})=\frac{p_{D,k}g_{k}(z_{k}^{p}\left|{\ddot{x}}_{k}^{\left(i\right)}\right.)}{\kappa_{k}(z_{k}^{p})+C_{k}(z_{k}^{p})}$$

(60) $$C_{k}(z_{k}^{p})=\sum_{j=1}^{L_{k-1}+J_{k}}p_{D,k}g_{k}(z_{k}^{p}\left|{\ddot{x}}_{k}^{\left(i\right)}\right.)\omega_{k\left|k-1\right.}^{\left(i\right)}$$

### 3.4. The Modification of Particles Weight

The estimate of target number relies on the updated weights of particles in the SMC-PHD filter. In our paper, the weight is updated by Equation (17) for each particle. The likelihood function is $g_{k}(z\left|{\ddot{x}}_{k}\right.)=\mathrm{St}(z;h(x_{k}^{\left(i\right)}),R_{k}^{\left(i\right)},\nu_{k}^{\left(i\right)})$, and the updated weight of the *i*th particle is given by

(61) $$\omega_{k}^{\left(i\right)}=(1-p_{D,k})\omega_{k\left|k-1\right.}^{\left(i\right)}+\sum_{p=1}^{\left|Z_{k}\right|}g_{k}^{p}({\ddot{x}}_{k}^{\left(i\right)})\omega_{k\left|k-1\right.}^{\left(i\right)}\propto \mathrm{St}(z_{k}^{p};h(x_{k}^{\left(i\right)}),R_{k}^{\left(i\right)},\nu_{k}^{\left(i\right)})$$

**Figure 1:**
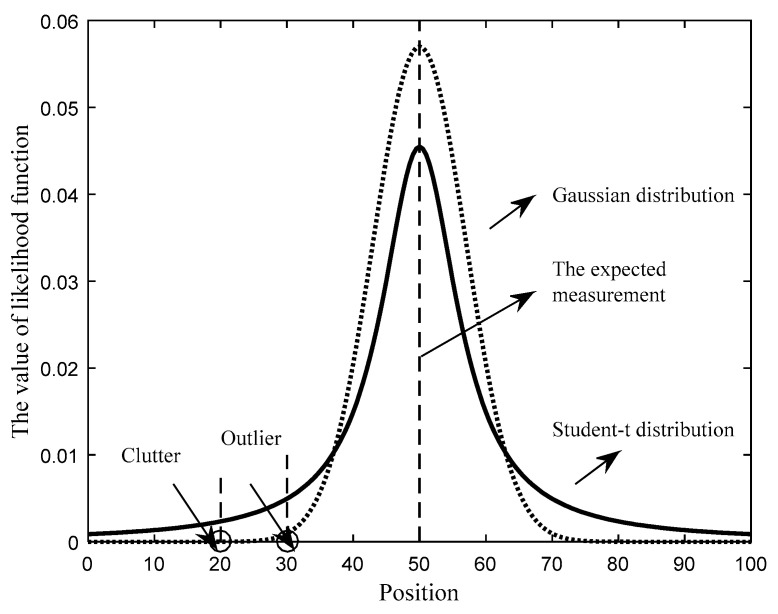
The illustration of measurement likelihood function.

Figure 1 gives the illustration of the measurement likelihood function. We can see that the Student-t distribution has a heavy-tailed characteristic and it can deal with outliers well by increasing the weights of outliers. However, the weight of clutter will increase at the same time. Hence, some clutter may be regarded as target-generated measurements, resulting in the overestimate of the target number at the current time.

To solve this problem, a strategy is adopted to modify the updated weight in this subsection. In the PHD filter, one target can produce at most one measurement and only one measurement can be attributed to one target per time step [4], named as the ‘one-to-one’ assumption. For each particle, the normalized measurement likelihood value $g_{k}^{p}({\ddot{x}}_{k}^{\left(i\right)})$ can be obtained by Equations (59) and (60). Let $J=\{p=1,\ldots ,\left|Z_{k}\right|\left|g_{k}^{p}({\ddot{x}}_{k}^{\left(i\right)})\geq \sigma \right.\}$, σ is a preset threshold. If $\left|J\right|\geq 2$, it indicates there might be multiple measurements associated with particle $x_{k}^{\left(i\right)}$. In this case, the $g_{k}^{p}({\ddot{x}}_{k}^{\left(i\right)})$ is modified as

(62) $${\tilde{g}}_{k}^{p}({\ddot{x}}_{k}^{\left(i\right)})=\left\{\begin{array}{c} g_{k}^{p}({\ddot{x}}_{k}^{\left(i\right)}),p=l\;\;or\;\;p\notin J\\ g_{k}^{q}({\ddot{x}}_{k}^{\left(i\right)}),p\neq l\;\;\&\;\;p\in J \end{array}\right.$$

where $q=\underset{p\in I}{\arg\min\;}g_{k}^{p}({\ddot{x}}_{k}^{\left(i\right)})$, & is a logic and operation and or is a logic or operation. In our paper, the measurement with the largest normalized likelihood value is regarded as the corresponding measurement generated from the target corresponding to particle $x_{k}^{\left(i\right)}$, and other measurements are regarded as clutter. The proposed strategy retains the maximum likelihood value and penalizes the other larger likelihood value to hold the ‘one-to-one’ assumption.

Then the modified weight can be expressed as

(63) $$\omega_{k}^{\left(i\right)}=(1-p_{D,k})\omega_{k\left|k-1\right.}^{\left(i\right)}+\sum_{p=1}^{\left|Z_{k}\right|}{\tilde{g}}_{k}^{p}({\ddot{x}}_{k}^{\left(i\right)})\omega_{k\left|k-1\right.}^{\left(i\right)}$$

The robust SMC-PHD filter is summarized in Algorithm 1.

**Remark** **1:** *Although the heavy-tailed characteristic of the Student-t distribution can deal with outliers, the target might still miss when the weights of outliers are lower than the extracted threshold and the target number would be underestimated. In our paper, the weight modification of the particles is mainly aimed to deal with the target number overestimate problem caused by clutter at the current time.*

**Algorithm 1.** The robust SMC-PHD filter.Step 1 Prediction for $i=1,2,\ldots ,L_{k-1}$    $x_{k}^{\left(i\right)}∼q_{k}(\cdot \left|x_{k-1}^{\left(i\right)},Z_{k}\right.),\omega_{k\left|k-1\right.}^{\left(i\right)}∼\omega_{k-1}^{\left(i\right)}\frac{p_{S,k}f_{k\left|k-1\right.}(x_{k}^{\left(i\right)}\left|x_{k-1}^{\left(i\right)}\right.)}{q_{k}(x_{k}^{\left(i\right)}\left|x_{k-1}^{\left(i\right)},Z_{k}\right.)}$    $\begin{array}{l} \upsilon_{k\left|k-1\right.}^{\left(i\right)}=\rho (\upsilon_{k-1}^{\left(i\right)}-d-1)+d+1,V_{k\left|k-1\right.}^{\left(i\right)}=\rho V_{k-1}^{\left(i\right)}\\ \alpha_{k\left|k-1\right.}^{\left(i\right)}=\rho \alpha_{k-1}^{\left(i\right)},\beta_{k\left|k-1\right.}^{\left(i\right)}=\rho \beta_{k-1}^{\left(i\right)} \end{array}$ end for for $i=L_{k-1}+1,L_{k-1}+2,\ldots ,L_{k-1}+J_{k}$    $x_{k}^{\left(i\right)}∼p_{k}(\cdot \left|Z_{k}\right.),\omega_{k\left|k-1\right.}^{\left(i\right)}∼\frac{1}{J_{k}}\frac{\gamma_{k}(x_{k}^{\left(i\right)})}{p_{k}(x_{k}^{\left(i\right)}\left|Z_{k}\right.)}$    $\upsilon_{k\left|k-1\right.}^{\left(i\right)}=\upsilon_{\gamma ,k}^{\left(i\right)},V_{k\left|k-1\right.}^{\left(i\right)}=V_{\gamma ,k}^{\left(i\right)}$    $\alpha_{k\left|k-1\right.}^{\left(i\right)}=\alpha_{\gamma ,k}^{\left(i\right)},\beta_{k\left|k-1\right.}^{\left(i\right)}=\beta_{\gamma ,k}^{\left(i\right)}$ end forStep 2 Update For $i=1,2,\ldots ,L_{k-1}+J_{k}$        $\begin{array}{l} R_{k}^{\left(i\right)}=V_{k\left|k-1\right.}^{\left(i\right)}/\left(\upsilon_{k\left|k-1\right.}^{\left(i\right)}-d-1\right),\nu_{k}^{\left(i\right)}=\alpha_{k\left|k-1\right.}^{\left(i\right)}/\beta_{k\left|k-1\right.}^{\left(i\right)}\\ g_{k}(z\left|{\ddot{x}}_{k}^{\left(i\right)}\right.)=\mathrm{St}(z;h(x_{k}^{\left(i\right)}),R_{k}^{\left(i\right)},\nu_{k}^{\left(i\right)}) \end{array}$        $\begin{array}{l} g_{k}^{p}({\ddot{x}}_{k}^{\left(i\right)})=\frac{p_{D,k}g_{k}(z_{k}^{p}\left|{\ddot{x}}_{k}^{\left(i\right)}\right.)}{\kappa_{k}(z_{k}^{p})+C_{k}(z_{k}^{p})}\\ C_{k}(z_{k}^{p})=\sum_{j=1}^{L_{k-1}+J_{k}}p_{D,k}g_{k}(z_{k}^{p}\left|{\ddot{x}}_{k}^{\left(i\right)}\right.)\omega_{k\left|k-1\right.}^{\left(i\right)} \end{array}$        $I=\left\{1,\ldots ,\left|Z_{k}\right|\right\},J=\left\{p=1,\ldots ,\left|Z_{k}\right|\left|g_{k}^{p}({\ddot{x}}_{k}^{\left(i\right)})\geq \sigma \right.\right\}$        $l=\underset{p\in I}{\arg\max}g_{k}^{p}({\ddot{x}}_{k}^{\left(i\right)}),q=\underset{p\in I}{\arg\min}g_{k}^{p}({\ddot{x}}_{k}^{\left(i\right)})$        ${\tilde{g}}_{k}^{p}({\ddot{x}}_{k}^{\left(i\right)})=\left\{\begin{array}{c} g_{k}^{p}({\ddot{x}}_{k}^{\left(i\right)}),p=l\;\;or\;\;p\notin J\\ g_{k}^{q}({\ddot{x}}_{k}^{\left(i\right)}),p\neq l\;\;\&\;\;p\in J \end{array}\right.$        $\omega_{k}^{\left(i\right)}=(1-p_{D,k})\omega_{k\left|k-1\right.}^{\left(i\right)}+\sum_{p=1}^{\left|Z_{k}\right|}{\tilde{g}}_{k}^{p}({\ddot{x}}_{k}^{\left(i\right)})\omega_{k\left|k-1\right.}^{\left(i\right)}$ Perform iteration initialization, set the iteration number τ, and update the parameters of measurement noise by Equations (49)–(60). end forStep 3 Resampling Step 4 State extraction    Step 3 and step 4 are the same as that in the standard SMC-PHD filter.

## 4. Simulation Results

To reveal the performance of the proposed robust SMC-PHD (RSMC-PHD) filter, we compare the Gaussian (Normal) Gamma inverse Wishart Gamma PHD (NGIWG-PHD) filter [23] and the SMC-PHD filter [6] with the proposed filter. For each simulation, 100 Monte Carlo trials are performed. The target number and optimal sub-pattern assignment (OSPA) [28] distance are chosen as the metric, where the parameters of OSPA are p=2 and c=100.

### 4.1. The Choice of Threshold σ

In Section 3.4, threshold σ is utilized to distinguish the target-generated measurements from clutter. In the SMC-PHD filter, the underlying target relies on the updated weight of each particle, and the likelihood value in Equation (59) can directly reflect the contribution of a given measurement or clutter to the underlying target. In most cases, the target-generated measurements usually have the largest likelihood value for each particle. If σ is larger, some clutter might be still regarded as target-generated measurements, and the overestimate of the target number cannot be solved. By contrast, if σ is smaller, the likelihood values of some clutter which contribute a small proportion to the updated weight will be modified. However, this modification will have no obvious effect on the updated weight of the particle except for unnecessary complexity. As we all know, $N_{particle}$ is the number of particles for each expected target in the SMC-PHD filter and it is used to extract the weighted particles to approximate the posterior intensity. To some extent, $1/N_{particle}$ can approximately reflect the average contribution of each particle for an underlying target. Therefore, we choose $\sigma =1/N_{particle}$ as the threshold.

### 4.2. Linear Scenario

The target state of each target is $x_{k}={\left[x_{k},{\dot{x}}_{k},y_{k},{\dot{y}}_{k}\right]}_{}^{T}$, consisting of the position ${\left[x_{k},y_{k}\right]}^{T}$ and velocity ${\left[{\dot{x}}_{k},{\dot{y}}_{k}\right]}_{}^{T}$. The target motion model is given by

(64) $$x_{k}=Fx_{k-1}+Gw_{k}$$

where

(65) $$F=\left[\begin{array}{cccc} 1 & \Delta & 0 & 0\\ 0 & 1 & 0 & 0\\ 0 & 0 & 1 & \Delta \\ 0 & 0 & 0 & 1 \end{array}\right]$$

(66) $$G={\left[\begin{array}{cccc} \Delta & 1 & 0 & 0\\ 0 & 0 & \Delta & 1 \end{array}\right]}^{T}$$

$w_{k}∼N(\cdot ;0_{2},Q_{k})$, $Q_{k}=\mathrm{diag}({\left[\sigma_{w}^{2},\sigma_{w}^{2}\right]}^{T})$, $\sigma_{w}=5\;{\;m/s}^{2}$ is the standard deviation of the process noise, Δ=1 s is the sampling period.

The observation model is formulated by

(67) $$z_{k}=Hx_{k}+\varepsilon_{k}$$

where

(68) $$H=\left[\begin{array}{cccc} 1 & 0 & 0 & 0\\ 0 & 0 & 1 & 0 \end{array}\right]$$

The heavy-tailed measurement noise corrupted by outliers is represented as [21,23]

(69) $$\varepsilon_{k}∼\left\{\begin{array}{l} N(\varepsilon_{k};0_{2},R_{0})\;\;\;\;\;\;\;\;\;\;1-\eta \\ N(\varepsilon_{k};0_{2},R_{n})\;\;\;\;\;\;\;\;\;\;\;\;\;\eta \end{array}\right.$$

where η denotes the probability of the measurement noise outlier. $R_{0}=\mathrm{diag}({\left[10^{2},10^{2}\right]}^{T})$ is the nominal noise variance, and $R_{n}=20R_{0}$.

The model of the birth target is a Poisson RFS with intensity

(70) $$\gamma_{k}(x)=\sum_{j=1}^{3}0.1N(x;m_{\gamma }^{\left(j\right)},P_{\gamma })$$

where $m_{\gamma }^{\left(1\right)}={\left[1200,0,-400,0\right]}^{T}$, $m_{\gamma }^{\left(2\right)}={\left[600,0,700,0\right]}^{T}$, $m_{\gamma }^{\left(3\right)}={\left[600,0,-300,0\right]}^{T}$,$P_{\gamma }=\mathrm{diag}({\left[100,100,100,100\right]}^{T})$. The survival probability is $p_{S,k}=0.99$ and the detection probability is $p_{D,k}=0.98$. The surveillance region is [0,2000]   m×[−1000,1000]   m. The clutter is uniformly distributed over the surveillance region, and the average clutter number is λ=10. For the particles of birth targets, $V_{\gamma ,k}^{\left(i\right)}=\mathrm{diag}({\left[10^{2},10^{2}\right]}^{T})$, $\upsilon_{\gamma ,k}^{\left(i\right)}=4$, $\alpha_{\gamma ,k}^{\left(i\right)}=3$, $\beta_{\gamma ,k}^{\left(i\right)}=1$. The number of iteration is τ=5, the decreasing factor is ρ=0.98, and the particles number is $N_{particle}=800$.

**Figure 2:**
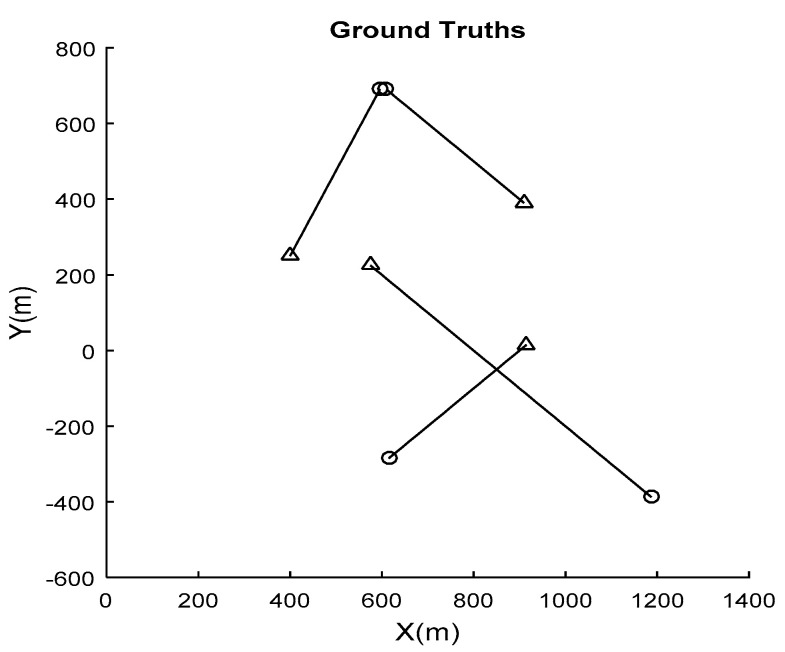
Trajectories of the targets.

The trajectories of the targets are shown in Figure 2, where circles are the beginning positions and triangles are the ending positions.

**Figure 3:**
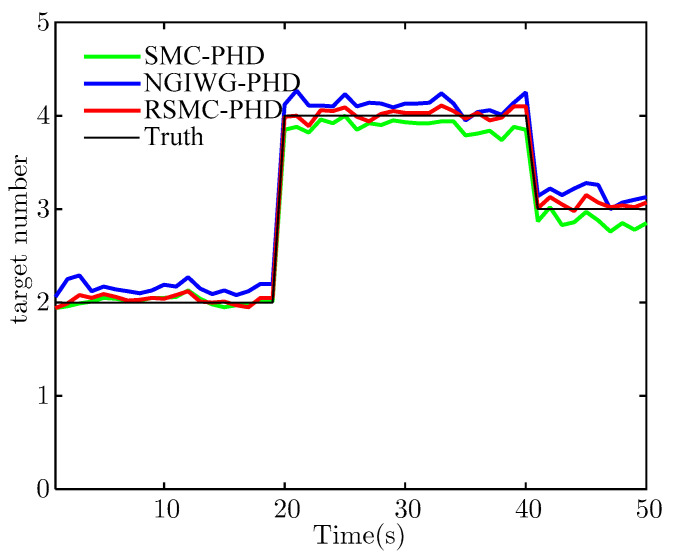
The estimate of target number.

**Figure 4:**
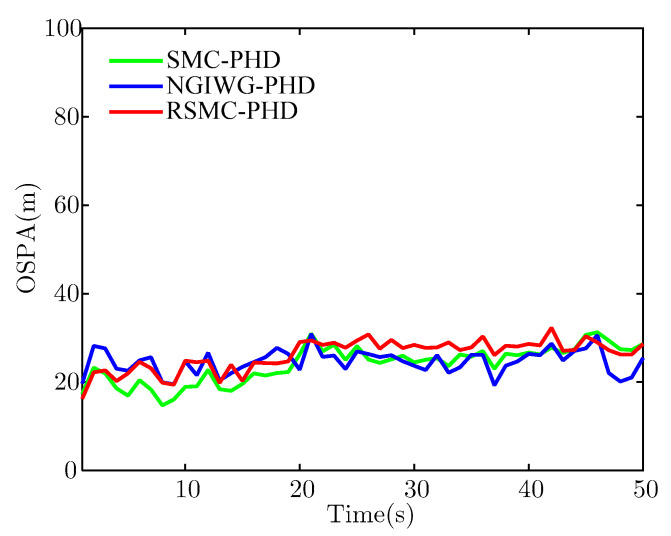
The estimate of OSPA.

To illustrate the performance of the proposed filter under the Gaussian measurement noise, η is set to 0. Figure 3 and Figure 4 show the estimate of target number and OSPA distance of the three filters, respectively. From Figure 3, we can see that all the filters achieve unbiased estimate of the target number and have a comparable estimate accuracy under Gaussian measurement noise. This is because the SMC-PHD filter utilizes the true measurement noise covariance. For the NGIWG-PHD filter and the RSMC-PHD filter, the DOF and scale matrix of the Student-t distribution can be adjusted automatically and the target tracking under Gaussian measurement noise realized. Figure 4 shows the OSPA distance of the three filters. Obviously, all the filters have an approximate OSPA distance.

To verify the multi-tracking performance under heavy-tailed measurement noise, η is set to 0.2. The remaining parameters are the same as above.

**Figure 5:**
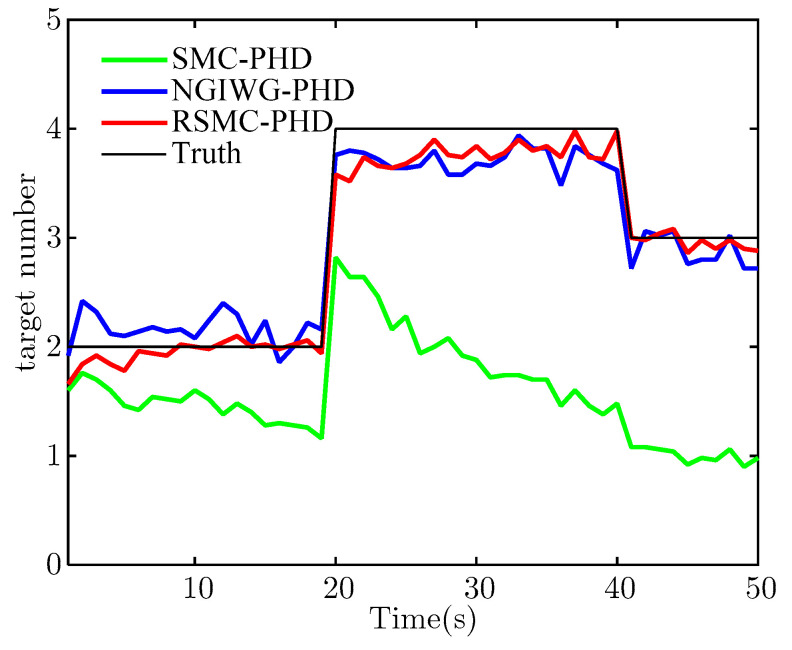
The estimate of target number.

**Figure 6:**
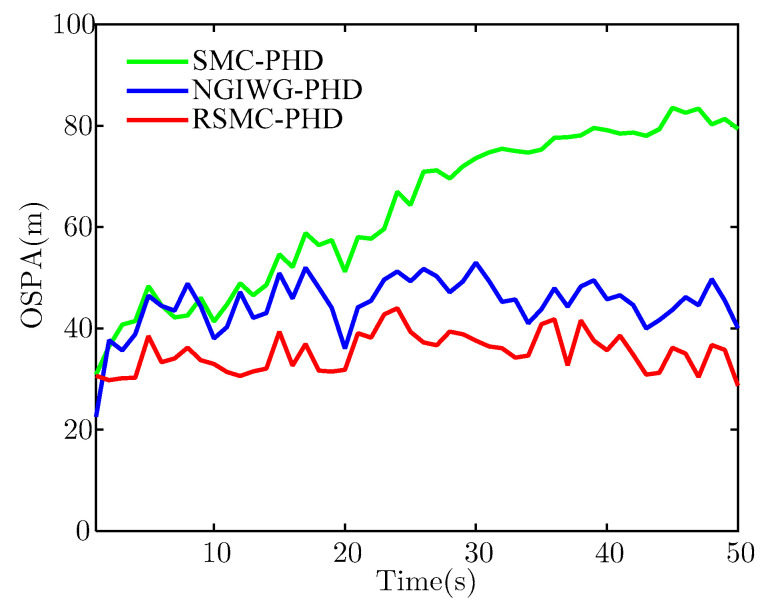
The estimate of OSPA.

Figure 5 and Figure 6 show the estimate of target number and OSPA distance of the three filters, respectively. Under heavy-tailed measurement noise, the SMC-PHD filter will diverge because the assumed measurement noise is not matched with the true measurement noise. For the NGIWG-PHD filter and the RSMC-PHD filter, the two filters will produce a weight which is non-negligible for filter update when there are outliers in measurements. Thus, the estimate accuracy of the target number of the NGIWG-PHD filter and the RSMC-PHD filter is higher than that of the SMC-PHD filter. From Figure 6, it can be observed that the RSMC-PHD filter has the best performance on OSPA distance.

**Figure 7:**
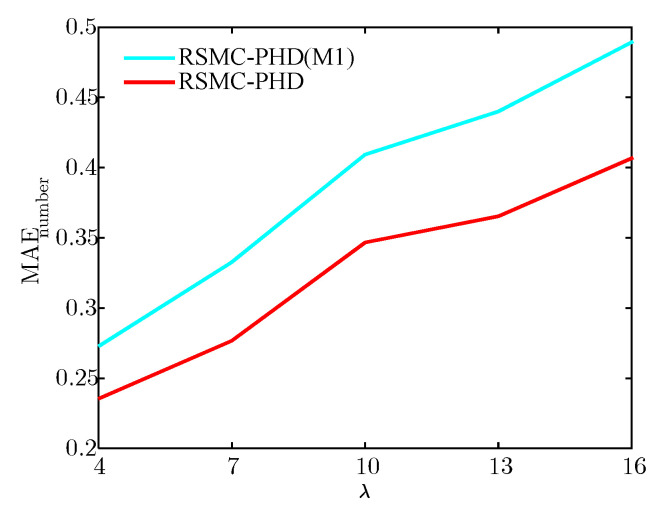
The MAE estimate of target number.

**Figure 8:**
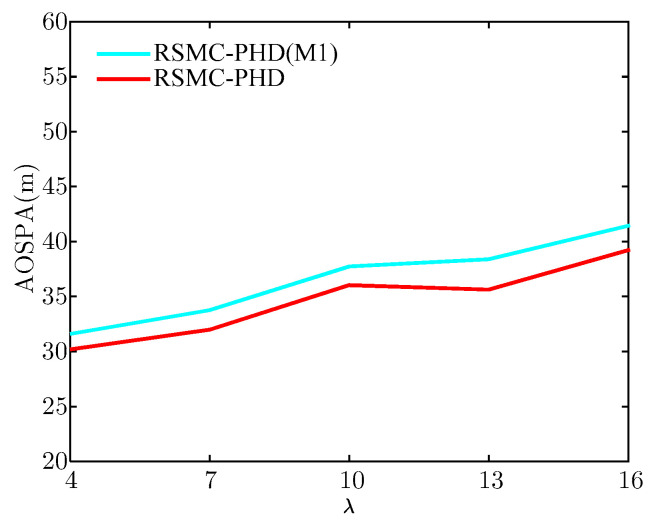
The estimate of AOSPA.

To verify the validity of the weight modification method, we compare the proposed RSMC-PHD filter with the proposed robust SMC-PHD filter without weight modification (RSMC-PHD(M1)). Figure 7 and Figure 8 show the mean absolute error (MAE) of the target number and the average OSPA (AOSPA) distance for the RSMC-PHD(M1) filter and the RSMC-PHD filter under different clutter number. The MAE of the target number and AOSPA are given by

(71) $${\mathrm{MAE}}_{\mathrm{number}}=\frac{1}{M_{C}T}\sum_{j=1}^{M_{C}}\sum_{k=1}^{T}\left|N_{k}^{j}-{\hat{N}}_{k}^{j}\right|$$

(72) $$\mathrm{AOSPA}=\frac{1}{M_{C}T}\sum_{j=1}^{M_{C}}\sum_{k=1}^{T}{\mathrm{OSPA}}_{k}^{j}$$

where $M_{C}$ is the number of Monte Carle trials, T is the simulation tracking time, $N_{k}^{j}$ and ${\hat{N}}_{k}^{j}$ are the true and the estimated target numbers at time k in the *j*th Monte Carle trial, ${\mathrm{OSPA}}_{k}^{j}$ is the corresponding OSPA distance.

Figure 7 illustrates that the MAE estimate of target number for the RSMC-PHD filter is lower than that of the RSMC-PHD(M1) filter. The more the clutter number, the higher is the probability that the clutter is regarded as the target-generated measurement, resulting in an overestimate of the target number at the current time. From Figure 8, it can be seen that the RSMC-PHD filter also outperforms the RSMC-PHD(M1) filter on AOSPA distance.

### 4.3. Nonlinear Scenario

To further evaluate the performance of the proposed filter, we consider a nonlinear scenario. For the NGIWG-PHD filter, EKF is used to handle nonlinearity.

The target state transition function is given by

(73) $$x_{k}=F_{ct}x_{k-1}+G_{ct}w_{k}$$

where

(74) $$F_{ct}=\left[\begin{array}{ccccc} 1 & \sin\omega \Delta /\omega & 0 & -\left(1-\cos\omega \Delta \right)/\omega & 0\\ 0 & \cos\omega \Delta & 0 & -\left(1-\cos\omega \Delta \right)/\omega & 0\\ 0 & \left(1-\cos\omega \Delta \right)/\omega & 1 & \sin\omega \Delta /\omega & 0\\ 0 & \sin\omega \Delta /\omega & 0 & \cos\omega \Delta & 0\\ 0 & 0 & 0 & 0 & 1 \end{array}\right]$$

(75) $$G_{ct}={\left[\begin{array}{ccccc} \Delta^{2}/2 & \Delta & 0 & 0 & 0\\ 0 & 0 & \Delta^{2}/2 & \Delta & 0\\ 0 & 0 & 0 & 0 & 1 \end{array}\right]}^{T}$$

The target state is $x_{k}={\left[x_{k},{\dot{x}}_{k},y_{k},{\dot{y}}_{k},\omega \right]}^{T}$, ω is the turn rate, $w_{k}∼N(\cdot ;0_{2},Q_{k})$, $Q_{k}=\mathrm{diag}({\left[\sigma_{x}^{2},\sigma_{y}^{2},\sigma_{\omega }^{2}\right]}^{T})$, $\sigma_{x}^{}=\sigma_{y}^{}=5\;{\;m/s}^{2}$, $\sigma_{\omega }^{}=5(\pi /180)\;\;\mathrm{rad}/s$, Δ=1  s.

The observation model is formulated by

(76) $$z_{k}=\left[\begin{array}{c} \sqrt{x_{k}^{2}+y_{k}^{2}}\\ \arctan(y_{k}/x_{k}) \end{array}\right]+\varepsilon_{k}$$

Assume the nominal measurement noise variance is $R_{0}=\mathrm{diag}({\left[10^{2},{\left(\pi /180\;\right)}^{2}\right]}^{T})$, and the true measurement noise is represented as (69) with $R_{n}=20R_{0}$ and η=0.2.

The model of birth target is a Poisson RFS with intensity

(77) $$\gamma_{k}(x)=\sum_{j=1}^{3}0.02N(x;m_{\gamma }^{\left(j\right)},P_{\gamma })$$

where $P_{\gamma }=\mathrm{diag}({\left[900,900,900,900,{(6\times \pi /180)}^{2}\right]}^{T})$, $m_{\gamma }^{\left(1\right)}={\left[-400,0,600,0,0\right]}^{T}$, $m_{\gamma }^{\left(2\right)}={\left[-250,0,250,0,0\right]}^{T}$, and $m_{\gamma }^{\left(3\right)}={\left[250,0,450,0,0\right]}^{T}$. The surveillance region is $\left[0,\pi \right]\mathrm{rad}\times \left[0,1500\right]m$. The clutter is uniformly distributed over the surveillance region and the average clutter number is λ=10. For the particles of birth targets,$V_{\gamma ,k}^{\left(i\right)}=\mathrm{diag}({\left[10^{2},{\left(\pi /180\;\right)}^{2}\right]}^{T})$, $\upsilon_{\gamma ,k}^{\left(i\right)}=4$, $\alpha_{\gamma ,k}^{\left(i\right)}=3$, $\beta_{\gamma ,k}^{\left(i\right)}=1$. The remaining parameters are the same as that in Section 4.2.

**Figure 9:**
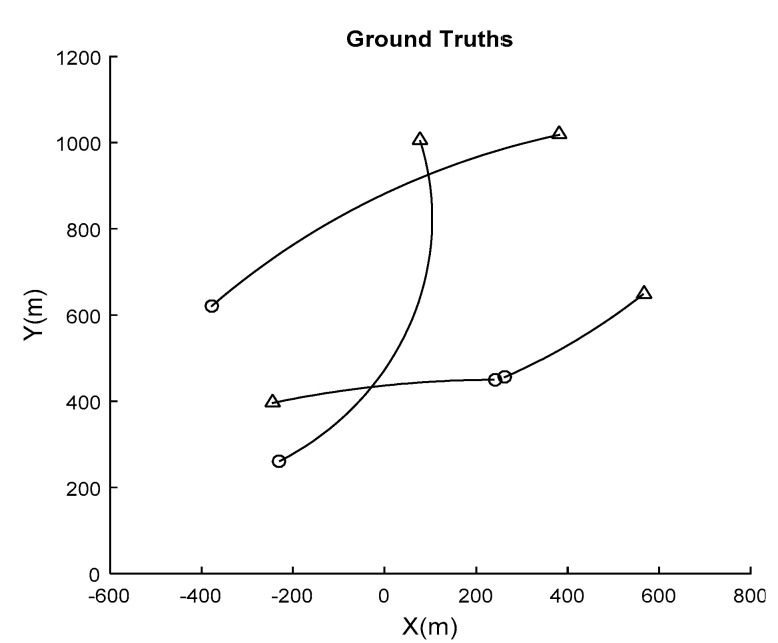
Trajectories of the targets.

The trajectories of the targets are shown in Figure 9, where circles are the beginning positions and triangles are the ending positions.

**Figure 10:**
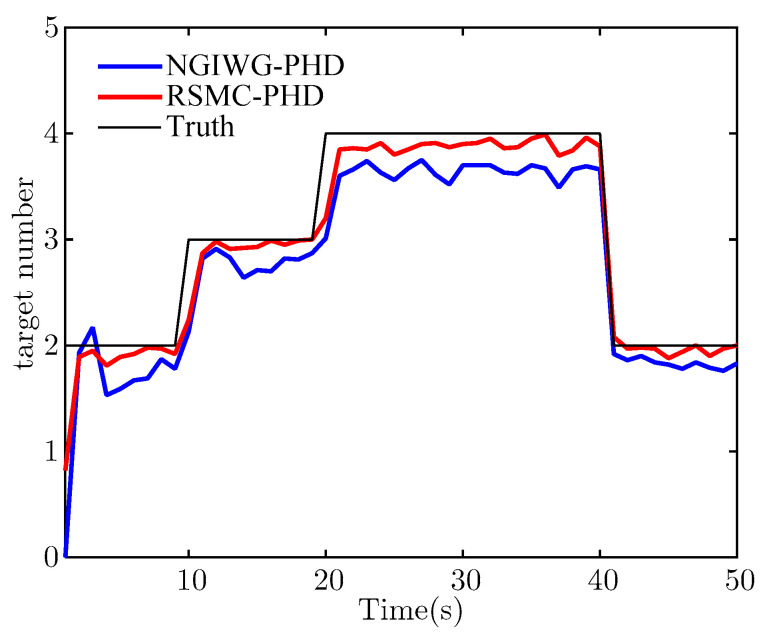
The estimate of target number.

**Figure 11:**
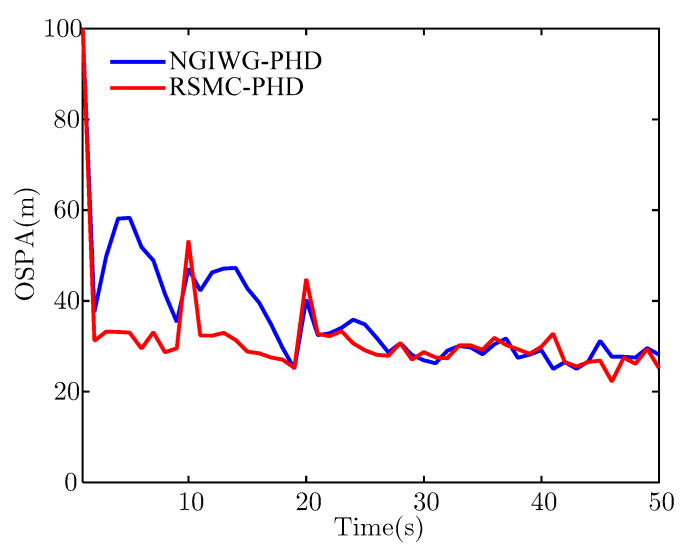
The estimate of OSPA.

Figure 10 and Figure 11 show the estimate of target number and OSPA distance for the two filters, respectively. It indicates that the NGIWG-PHD filter performs worse in the nonlinear system. This is because the NGIWG-PHD filter uses EKF to linearize the nonlinear model, and the estimate error of the target state will become large. Under the influence of heavy-tailed measurement noise, the target will miss and the target number will be underestimated. On the contrary, the RSMC-PHD filter is not subject to nonlinear constraint, and has a better performance in the nonlinear system.

## 5. Conclusions

In this paper, a robust SMC-PHD filter with unknown heavy-tailed measurement noise is proposed. The proposed filter uses the Student-t distribution to model the unknown measurement noise. The IW distribution is used to model the unknown scale matrix parameter. Meanwhile, the Gamma distribution is used to model the unknown DOF parameter. Then, the unknown noise parameters are updated by the VB technique within the SMC-PHD filter framework. In addition, a strategy is applied to solve the overestimate of the target number induced by the Student-t distribution. Simulation results under the linear system and nonlinear system show that the proposed filter has a better performance than the compared filters with the unknown heavy-tailed measurement noise, especially in a nonlinear system.

## Data Availability

Not applicable.
